# Analysis of rare Parkinson’s disease variants in millions of people

**DOI:** 10.21203/rs.3.rs-2743857/v1

**Published:** 2023-04-10

**Authors:** Vanessa Pitz, Mary Makarious, Sara Bandrés-Ciga, Hirotaka Iwaki, Andrew Singleton, Mike Nalls, Karl Heilbron, Cornelis Blauwendraat

**Affiliations:** NIA/NIH; NIH; Laboratory of Neurogenetics National Institute on Aging; Data Tecnica International; National Institute on Aging (NIA), NIH; National Institutes of Health; 23andMe Inc; NIH

**Keywords:** Parkinson’s, rare variants, genetics

## Abstract

**Objective:**

Although many rare variants have been reportedly associated with Parkinson’s disease (PD), many have not been replicated or have failed to replicate. Here, we conduct a large-scale replication of rare PD variants.

**Methods:**

We assessed a total of 27,590 PD cases, 6,701 PD proxies, and 3,106,080 controls from three data sets: 23andMe, Inc., UK Biobank, and AMP-PD. Based on well-known PD genes, 834 variants of interest were selected from the ClinVar annotated 23andMe dataset. We performed a meta-analysis using summary statistics of all three studies.

**Results:**

The meta-analysis resulted in 11 significant variants after Bonferroni correction, including variants in *GBA1* and *LRRK2*. At least 9 previously reported pathogenic or risk variants for PD did not pass Bonferroni correction in this analysis.

**Conclusions:**

Here, we provide the largest rare variant meta-analysis to date, providing thorough information of variants confirmed, newly identified, or rebutted for their association with PD.

## Introduction

Over the past several decades, common and rare variants in multiple genes have been associated with Parkinson’s disease (PD). PD is a neurodegenerative disorder primarily affecting dopaminergic neurons in the substantia nigra, and is caused by a combination of aging, environmental factors, and genetics. Genetics account for a large proportion of PD risk (Blauwendraat, Nalls, and Singleton 2020; Bandres-Ciga et al. 2020). Several large-scale case-control genome-wide association studies (GWAS) have been performed and have identified 90 risk variants (Nalls et al. 2019), typically with moderate odds ratios (OR, mean = 1.24, range: 1.05–11.35). Many rare variants that have been associated with PD are considered either a strong risk factor (OR > 2) or a monogenic cause of disease. Pathogenic variants in *GBA1* and *LRRK2* are the most common high-risk genetic factors for PD, typically present in 1–10% of the PD population depending on genetic ancestry. Most other known pathogenic variants are very rare (allele frequency < 0.1%, e.g. damaging variants in *SNCA* and *PRKN*).

Typically, these rare variants are also associated with a slightly different PD phenotype, highlighting the importance of their research even more. Overall, more severe motor and non-motor symptoms are seen in *GBA1* variant carriers (Lesage et al. 2020; Brockmann et al. 2015; Stoker et al. 2020), a more rapid progression to dementia in *SNCA* carriers (Q. Wang et al. 2016; Meeus, Theuns, and Van Broeckhoven 2012; Fuchs et al. 2007), and very early onset and an akinesia-related phenotype in *PRKN* carriers (Khan et al. 2003). It is important to note that the status of some reported PD genes is debated, since most associations are reported without a replication cohort or in small, often biased single-case or family studies (Blauwendraat et al. 2018; Blauwendraat, Nalls, and Singleton 2020). Previously, we investigated the *SNCA* p.H50Q mutation, which was identified in a pathologically-proven PD case without family segregation (Proukakis et al. 2013). However, when assessing this variant in a large-scale study including over 10,000 cases and 10,000 controls, no association was identified (Blauwendraat et al. 2018). These data do not support a pathogenic role for SNCA p.H50Q despite functional evidence supporting a potential role in disease (Boyer et al. 2019). This potentially also applies to variants in other genes including *DNAJC13, EIF4G1, GIGYF2, HTRA2, LRP10, TMEM230* and *UCLH1*, all of which have been categorized previously as low-confidence PD genes (Blauwendraat, Nalls, and Singleton 2020). A robust assessment of high-risk and causal PD variants will be incredibly valuable for the global PD community from laboratory researchers to genetic counselors.

Here we tested PD mutations from the ClinVar database for association with PD in three large case-control cohorts (23andMe, Inc., UK Biobank, and AMP-PD) totalling over 3 million individuals (27,590 cases, 6,701 proxies, and 3,106,080 controls). The large number of participants in this study is an opportunity to assess rare variants in PD more reliably, which has not previously been possible to this extent. Our goal was to create lists of high-confidence and low-confidence PD variants. This work improves our understanding of the clinical relevance of these variants, which can then be used to improve genetic testing in patients.

## Methods

### Gene and variant selection

**Figure 1:**
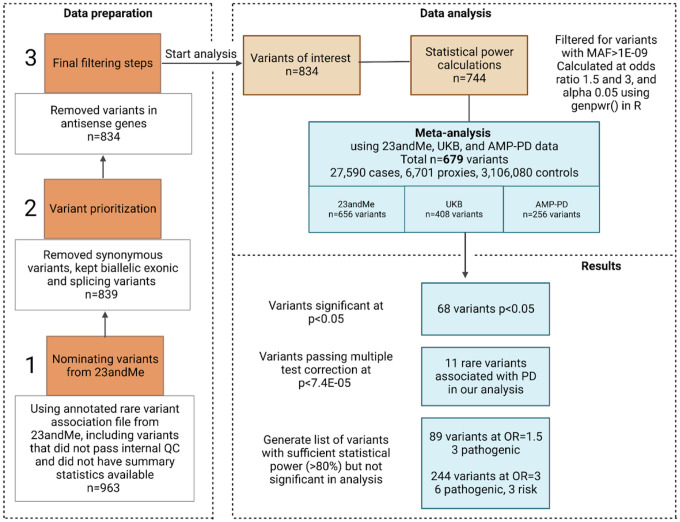
Analysis flowchart *Data preparation:* Variant selection was based on an annotated 23andMe rare variant association file, including variants that passed or failed internal quality control steps, resulting in some variants not having summary statistics available but could still be used for variant selection in other data sets. Data were filtered to remove synonymous variants and to keep biallelic exonic, and splicing variants. *Data analysis:* 744 variants of interest were used to calculate statistical power for each variant at odds ratio 1.5 and 3, and alpha 0.05. 656 variants had summary statistics available in 23andMe data and were analyzed individually but also as part of a meta-analysis, using 23andMe variants found in UK Biobank and AMP-PD data. *Results:* Results were assessed for different significance thresholds, main results focused on variants passing Bonferroni correction but also variants that reached sufficient statistical power but were not associated with PD in this analysis. Figure created with BioRender.com.

For variant selection, we annotated PD GWAS summary statistics from 23andMe using ANNOVAR (K. Wang, Li, and Hakonarson 2010) including gene-based and ClinVar (version clinvar_20220320) annotation. We investigated variants with a minor allele frequency (MAF) of less than 5% to restrict our focus to rare variants while retaining known risk variants such as *GBA1* p.E365K. Only biallelic exonic or splicing variants related to “Parkinson’s disease” and/or “Lewy body dementia” were kept. We selected all coding variants from genes previously published in literature: *ATP13A2, DNAJC13, DNAJC6, EIF4G1, FBXO7, GBA1, GIGYF2, HTRA2, LRP10, LRRK2, PARK7, PINK1, PLA2G6, POLG, PRKN, SNCA, SYNJ1, TMEM230, UCHL1, VPS13C, VPS35* (Blauwendraat, Nalls, and Singleton 2020) and also removed “benign” and “likely benign” variants in the *GBA1* gene but also in genes identified in the keyword search “Parkinson’s disease” and “Lewy body dementia”. Synonymous, *PINK1*-antisense (AS), and *UCHL*-AS variants were also excluded from the dataset. The process of variant selection based on 23andMe data is summarized in Fig. 1.

### 23andMe Data

A rare variant association analysis was conducted using 3,090,507 unrelated people (3,065,473 controls, 25,034 PD cases). Data was processed as previously published in (Heilbron et al. 2021). In brief: related individuals were removed, defined as > 700cM that are identical-by-descent (~ 20% of the genome or approximately first cousins in an outbred population) (Henn et al. 2012). Ancestry composition was performed as previously reported (Durand et al., n.d.), and to minimize confounding by ancestry, only individuals with predominantly European ancestry were used.

Phased participant data were imputed using Minimac3. Throughout, structural variants and small indels were treated the same as SNPs. Association test results were computed by logistic regression assuming additive allelic effects. Covariates for age, sex, and the top five genetic principal components (PCs) were included to account for residual population structure, and indicators for genotype platforms to account for genotype batch effects. The association test p-value reported was computed using a likelihood ratio test. Genotyped SNPs were excluded that: 1) had a genotyping rate < 90%, 2) were only genotyped on the “v1” or “v2” 23andMe genotyping array, 3) were found on the mitochondrial chromosome or the Y-chromosome, 4) failed a test for parent-offspring transmission (p < 10^− 20^), 5) had an association with genotype date (p < 10^−50^ by ANOVA of SNP genotypes against a factor dividing genotyping date into 20 roughly equal-sized buckets), 6) had a large sex effect (ANOVA of SNP genotypes, r2 > 0.1), or 7) had probes matching multiple genomic positions in the reference genome. For tests using imputed data, we used the imputed dosages rather than best-guess genotypes. Imputed SNPs were excluded that: 1) had imputation r2 < 0.5 for individuals genotyped on the “v4” and “v5” arrays, or 2) had a significant batch effect between the “v4” and ”v5” genotyping arrays (p < 10^− 50^ by ANOVA of SNP dosage against genotyping array). Both genotyped and imputed SNPs were removed if: 1) available sample size was less than 20% of the total GWAS sample size, or 2) the logistic regression failed to converge (absolute value of the estimated log odds ratio or standard error > 10).

Participants provided informed consent and volunteered to participate in the research online, under a protocol approved by the external AAHRPP-accredited IRB, Ethical & Independent (E&I) Review Services. As of 2022, E&I Review Services is part of Salus IRB (https://www.versiticlinicaltrials.org/salusirb). The full GWAS summary statistics for the 23andMe discovery data set will be made available through 23andMe to qualified researchers under an agreement with 23andMe that protects the privacy of the 23andMe participants. Datasets will be made available at no cost for academic use. Please visit https://research.23andme.com/collaborate/#dataset-access/ for more information and to apply to access the data.

### AMP-PD and UK Biobank Data

Association results for the variants selected from the 23andMe dataset were generated from whole-genome sequencing data made available by the Accelerating Medicines Partnership - Parkinson’s disease Initiative (AMP-PD, https://amp-pd.org/) and the whole-exome sequencing data made available by the UK Biobank (https://www.ukbiobank.ac.uk/). Processing of these cohorts have been previously described elsewhere (Makarious et al. 2022). In brief, the AMP-PD datasets consist of unrelated individuals and publicly available cohorts only. After filtering and removing those recruited in a genetic study, the data set comprises a total of 4,007 people (2,197 males, 1,810 females) and 2,556 of those are controls and 1,451 are PD cases. The UK Biobank is a large-scale biomedical database containing genetic and health information from half a million participants (Bycroft et al. 2018). Similar filtering parameters were used for UK Biobank resulting in a total of 45,857 people (22,040 males, 23,817 females), of which 38,051 are controls, 1,105 cases, 6,033 individuals with a parent that is diagnosed with PD, and 668 individuals with a sibling that is diagnosed with PD (6,701 proxy cases). As previously described, Controls were filtered to exclude any individuals with an age of recruitment < 59 years, any reported nervous system disorders (Category 2406), a parent with PD or dementia (field codes: 20107 and 20110) and any reported neurological disorder (field codes: Dementia/42018, Vascular dementia/42022, FTD/42024, ALS/42028, Parkinsonism/42030, PD/42032, PSP/42034, MSA/42036) (Makarious et al. 2022).

### Statistical analyses

We used three different data sets, including summary statistics from 23andMe and sequencing data from AMP-PD and UK Biobank. All data used genome build GRCh38. PLINK (v1.9, (Purcell et al. 2007)) was used to extract the variants identified in 23andMe data from AMP-PD and UK Biobank. To generate the association files for AMP-PD and UK Biobank, we then used RVTests (Zhan et al. 2016) for single variant association testing, using sex, and principal components (PC) 1 to 5 as covariates for AMP-PD, excluding age since this is an age-matched cohort. Genetic sex, age at recruitment, Townsend score, and PC 1 to 5 were used as covariates for UK Biobank. We conducted a fixed-effect inverse variance-weighted meta-analysis with the summary statistics, using METAL (version 2020-05-05 (Willer, Li, and Abecasis 2010)). Results were annotated using ANNOVAR, refGene, avsnp150, and clinvar_20220320 (K. Wang, Li, and Hakonarson 2010). Forest plots were generated using the rmeta (version 3.0) and metafor (version 3.8–1) packages in R. Power calculations were conducted using the R (v. 3.6) package genpwr (version 1.0.4), a power and sample size calculator for genetic association studies which allows for misspecification of the model of genetic susceptibility (Moore, Jacobson, and Fingerlin 2019). This package allows the assessment of allele frequencies as low as 1E-9 at OR = 1.5, and as low as 1E-04 at OR = 3. We used an additive model with an alpha value of 0.05. Since we used an additive model, it is important to note that we had less power to detect recessive associations in our analysis.

## Results

### Variant selection and dataset details

We attempted to replicate 834 published associations between PD and protein-altering genetic variants (Fig. 1, see Methods). 609 (73%) of the ClinVar annotated variants had either no reported clinical significance, or had conflicting or uncertain interpretations. Of the remaining 225 variants, 133 variants (15.9%) were classified as pathogenic and/or likely pathogenic, followed by 84 variants (10.1%) classified as benign or likely benign variants. 6 variants (0.7%) were reported to be risk factors for PD, and 2 (0.2%) variants were reported to be either pathogenic/risk factors **(Supplementary Table 1A)**. The 834 variants were located in 32 genes including *GBA1* (n = 103), *VPS13C* (n = 101), and *LRRK2* (n = 97) **(Supplementary Table 2)**.

The list of 834 variants includes variants that did not pass 23andMe-internal QC and therefore had no summary statistics, but were still useful for variant selection in other studies. 282 (33.8%) variants were available in the AMP-PD dataset, 608 (72.9%) in UK Biobank, and 656 (78.7%) variants in 23andMe, resulting in a total of 679 unique variants. We focused on significant variants passing Bonferroni correction in each analysis, then generated a list of significant hits, calculating OR with a 95% confidence interval.

## Analysis

We conducted a meta-analysis using summary statistics generated by the single-variant association testing data from 23andMe, UK Biobank, and AMP-PD (Table 1). 23andMe data comprised 25,034 PD cases and 3,065,473 controls. Cases were 59.9% male with a mean age of 72.3 (± 10.9) years, controls were 43.5% male with a mean age of 50.1 (± 17.5) years. The UK Biobank dataset comprised 1,105 cases and 6,701 proxies. Cases and proxies were 45.6% male with an average age of 59.1 (± 7.1) years, 38,051 were controls (48.6% male) with a mean age of 64.1 (± 2.8) years. AMP-PD had 1,451 cases (63.7% male) with a mean age of 61.3 (± 10.2) years, and 2,556 controls (49.8% male) with a mean age of 70.7 (± 13.2) years. A summary of cohort demographics can be found in Table 1.

679 unique variants were analyzed in the meta-analysis, using 656 variants from 23andMe, 408 from UK Biobank, and 256 from AMP-PD. 249 (36.7%) variants were solely contributed by 23andMe, whereas 18 (2.7%) variants were only found in UK Biobank, and 1 (0.1%) variant was only found in AMP-PD (Supplementary Fig. 1). 68 variants passed the significance threshold of p < 0.05, and 11 variants passed Bonferroni correction of p < 7.4E-05, based on 0.05/679 (forest plots are provided in Supplementary Fig. 3). MAFs ranged from 4.0E-07 to 0.012. The results with their ClinVar designation comprised 4 pathogenic variants: *PRKN* p.R275 (rs34424986, OR = 1.3, 95% CI: 1.15–1.47). *GBA1* p.R296Q (rs78973108, OR = 3.7, 95% CI: 2.1–6.5), *GBA1* p.R502C (rs80356771, OR = 3.8, 95% CI: 2.7–5.4), and *LRRK2* p.R1441H (rs34995376, OR = 43.0, 95% CI: 13.8–133.9). 2 variants were pathogenic/risk factor: *GBA1* p.N409S (rs76763715, OR = 2.3, 95% CI: 2.0–2.5) and *LRRK2* p.G2019S (rs34637584, OR = 11.8, 95% CI: 10.5–13.2). The 5 remaining variants were of conflicting, uncertain, or unknown clinical significance: *GBA1* p.E365K (rs2230288, OR = 1.4, 95% CI: 1.3–1.5), *GBA1* p.T408M (rs75548401, OR = 1.5, 95% CI: 1.4–1.6), *LRRK2* p.L1795F (rs111910483, OR = 2.5, 95% CI: 1.6–3.8), *GBA1* p.D179H (rs147138516, OR = 3.3, 95% CI: 2.1–5.0), and *GBA1* p.S146L (rs758447515, OR = 1624, 95% CI: 57–45172).

**Figure 2:**
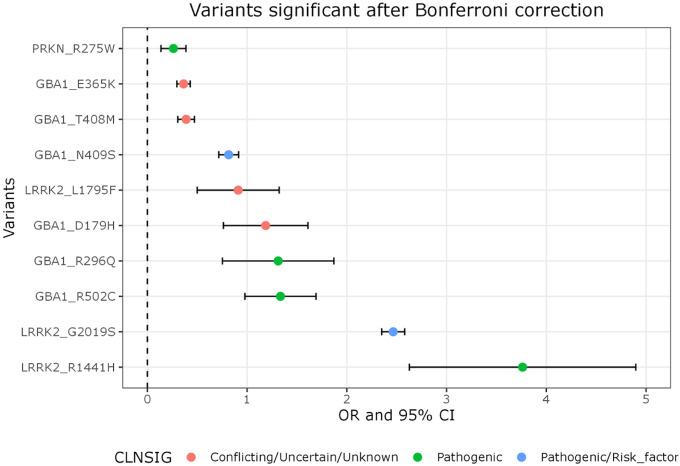
Variants passing Bonferroni correction Forest plot showing 11 variants passing Bonferroni correction in the meta-analysis. Data is based on OR (dot) and 95% CI (error bar) and for visualization purposes the x-axis is on a log scale. Colors indicate clinical significance of the variant: Conflicting/uncertain/unknown (red), pathogenic (green), and pathogenic/risk factor (blue) based on ClinVar annotations. Note: *GBA1* p.S146L (conflicting/uncertain/unknown clinical significance) also remained significant after multiple test correction, however with OR=1,624, 95% CI: 58–45,172, the statistics were too large to visualize in this plot.

Based on the ClinVar database, *GBA1* p.D179H and *GBA1* p.S146L were not previously reported to be associated with PD and it is worth noting that (as is expected for very rare variants with MAF < 1%) especially *GBA1* p.S146L had wide confidence intervals. *GBA1* p.S146L was identified in 0 cases, 3 proxy cases (individuals with a parent with PD), and 0 controls in UK Biobank; and did not pass 23andMe QC (Fig. 2, Table 2). Interestingly, *LRRK2* p.L1795F has been identified previously in a family with PD (Nichols et al. 2007), however no segregation was shown and further reports are lacking in the literature. This variant was identified in 2 cases and 0 controls in AMP-PD, shows a significant association in the 23andMe cohort (OR: 2.3, 95% CI: 1.57–3.61, P = 0.0003), but was not present in UKB.

### Power calculations of variants of interest

In addition to confirming previously identified variants and finding new associations, another aim of this work was to assess if we could successfully replicate PD-associated variants found in the literature. However, when working with such low allele frequencies, one of the main challenges is sufficient statistical power. This requires very large sample sizes to capture sufficient numbers of rare variant carriers for robust statistical estimation.

We conducted power calculations for all variants in the meta-analysis, accounting for the tool’s MAF limit of > 1E-09 (n = 669, **Supplementary table 8**) and had a special focus on variants with a p-value > 0.05 (n = 601) to assess whether we had > 80% statistical power to detect an association assuming OR = 1.5, alpha = 0.05, and an additive model. The parameters set for this calculation are generous, since an OR of 1.5 is insufficient to cause monogenic disease in affected individuals and we are not correcting for multiple testing. Therefore, under these parameters, any association that had > 80% power but was not statistically significant in our analysis is very likely to be a spurious association.

We reached 80% power for 82 variants at OR = 1.5 and alpha 0.05. 41 (50%) were of conflicting, uncertain or unknown clinical significance, 38 (46%) were benign, and 3 (3%) were pathogenic. The pathogenic variants were *GLUD2* p.S498A (rs9697983, OR: 1.02, 95%CI: 0.97–1.07), *POLG* p.A467T (rs113994095, OR: 1.03, 95%CI: 0.79–1.36), and *POLG* p.G737R (rs121918054, OR: 1.01, 95%CI: 0.84–1.22). *GLUD2* p.S498A is the only variant that has previously been associated with PD. *POLG* variants were associated with seizures, toe walking, progressive sclerosing poliodystrophy, and many other diagnoses. Since POLG mutations typically cause disease in autosomal recessive patterns the association here is not a surprise finding, however our results provide evidence against a pathogenic role of the *GLUD2* variant in PD.

When increasing the OR to 3, we reached 80% power for 231 variants. 171 (74%) were of conflicting, uncertain or unknown significance, 51 (22%) were benign, 5 (2%) were pathogenic, and 3 (1%) were risk variants. 3 pathogenic variants were the same as the ones at OR = 1.5, however, we additionally had sufficient power for PINK1 p.T313M (rs74315359, OR: 1.30, 95% CI: 0.64–2.64), SNCB p.P123H (rs104893937, OR: 1.25, 95% CI: 0.68–2.31), and *POLG* p.G848S (rs113994098, OR: 1.11, 95% CI: 0.59–2.11). The *SNCB* variant is associated with Lewy body dementia and *PINK1* p.T313M has previously been associated with PD. Similar to the POLG mutations, *PINK1* mutations cause disease in autosomal recessive patterns and therefore the lack of association here is not a surprise finding.

## Discussion

Here, we assessed the role of rare variants and their association with PD using several large case-control datasets of European ancestry. We conducted a single rare variant association analysis of PD vs controls, including 23andMe, UK Biobank and AMP-PD; totalling over 3 million individuals comprising 27,590 cases, 6,701 PD proxy cases, and 3,106,080 controls. We provide very robust evidence of 11 high risk and causal variants for PD disease development. Additionally, we provide evidence that a large number of variants that have previously been associated with PD are likely not associated with PD and should be treated with caution.

Our findings clearly confirm the prominent role of variants in *LRRK2* and *GBA1* on increased PD risk, in particular: *LRRK2* p.R1441H, p.L1795F and p.G2019S; and *GBA1* p.S146L, p.D179H, p.R296Q, p.E365K, p.T408M, p.N409S and p.R502C. All of these variants have odds ratios close to or over the threshold of 1.5, which is considered clinically more relevant (Szumilas 2010; Norton, Dowd, and Maciejewski 2018). Additionally, here we update the risk estimates for these variants with a more reliable confidence interval than previously reported. *LRRK2* p.L1795F is a lesser-known and studied variant compared to p.R1441H and p.G2019S, which are both well-known damaging variants. *LRRK2* p.L1795F is located in the C-terminal of ROC B region and has been reported in a family with PD, however segregation was not shown (Ghani et al. 2015). Here, we provide evidence for *LRRK2* p.L1795F as a genetic risk factor for PD with an estimated OR of 2.5. Interestingly, this variant was recently shown to have a functional effect providing more evidence for its pathogenicity (Kalogeropulou et al. 2022). *GBA1*, p.E365K, p.T408M and p.N409S all have been previously identified via GWAS or similar approaches (Nalls et al. 2019), with p.E365K and p.T408M being risk factors for PD, and p.N409S being a risk factor for PD with and additional association with Gaucher disease. The other *GBA1* variants (p.S146L, p.D179H, p.R296Q and p.R502C) are all associated with Gaucher disease in a biallelic state and were robustly associated with PD in this study. In the case of *GBA1* p.D179H, the association with PD was statistically evident with higher ORs and narrow 95% CIs: 23andMe reporting OR = 3.4 (95% CI: 2.2–5.2) and OR = 3.3 (95% CI:2.1–5.0) in the meta-analysis. However it is worth noting that this variant is often on the same haplotype as p.E365K (Pawliczek et al. 2018; den Heijer et al. 2020) and indeed when exploring AMP-PD data we also identified that all instances of the p.D179H allele are in cis (D’=1) with p.E365K.

Many variants, common or rare, are reported to be associated with PD, in fact the ClinVar database shows 4,320 results for the condition when searching for “Parkinson” as of January 1, 2023. To generate a list of variants that were not replicated previously in large case-control analyses, we assessed whether we had > 80% power at OR = 1.5 and 3, using a p-value threshold of p > 0.05. Most well-powered variants with meta-analysis p-value > 0.05 were of uncertain, unknown or unclear clinical significance, but quite a few were also previously associated with Parkinson’s (**Supplementary Table 3**). For example, 6 variants that were previously reported to be “pathogenic” or “likely pathogenic” did not show evidence of association with PD and therefore these variants should be treated with caution. Those variants were: *POLG* p.467T (rs113994095), p.G737R (rs121918054), and p.G848S (rs113994098); *GLUD2* p.S498A (rs9697983), *PINK1* p.T313M (rs74315359), and *SNCB* p.P123H (rs104893937). Interestingly, some of these variants always had very weak evidence for an association in previous publications but were still considered PD variants in the ClinVar database. The *PINK1* p.T313M variant was only found to be associated with PD in a single Saudi Arabian family (Chishti et al. 2006; Plaitakis et al. 2010). However, due to our study design, results regarding autosomal recessive genes should be interpreted carefully.

We acknowledge that our analysis comes with some limitations. First, very large sample sizes are required to study rare variants. Investigating variants with low frequencies often results in unreliable high odds ratios with wide confidence intervals, suggesting that the population parameter is not confidently predicted. For example the *GBA1* p.S146L variant (rs758447515, OR = 1624.7, 95% CI = 58–45172), whose generated statistics suggest that it plays a role in PD risk but will need to be further studied to determine more accurate risk estimates. Another example is *SNCA* p.A53T which is known to be causal for PD (Polymeropoulos et al. 1997), but given the extreme low frequency of this allele it was not found in AMP-PD or UK Biobank data, and had a p-value of 0.03 with a very wide risk estimate OR = 38.0, 95% CI = 2.82–510.12 in the 23andMe dataset. This clearly shows association but not enough carriers to pass multiple test correction. Additionally, some pathogenic variants passing significance at p < 0.05 but failing the multiple test correction were identified. *GBA1* p.V433LF (rs80356769, meta-analysis: P = 1.6E-04, OR = 4.2, 95% CI = 1.98–8.8) was present in ClinVar as a pathogenic PD variant, but also the previously linked *LRRK2* variant p.I2020T (rs35870237, P = 0.0004, OR = 21.4, 95% CI = 3.97–115.5) is considered pathogenic based on biological evidence (Ray et al. 2014). *LRRK2* p.I2020T is associated with autosomal dominant PD (Paisán-Ruíz et al. 2004; Zimprich et al. 2004). These variants showcase the complexities of rare variant analysis and although we used the largest sample size for PD yet, it still shows the limited power we have to identify robust risk estimates for very rare variants.

Second, an important aspect of rare and causal variants is the allele-dosage effect, since recessive genes are only causal in a homozygous or compound heterozygous state, which is especially important for PD since several genes are known to only be causal in a recessive state. Most of our statistical models used here are based on additive effects and since only summary level data was available from 23andMe, we cannot accurately report results on autosomal recessive genes such as *PRKN* and *PINK1*. To highlight this further the *PRKN* p.R275W (rs34424986) variant is known to increase the risk for PD in a homozygous or compound heterozygous state (Lubbe et al. 2021). In our analysis this particular variant also made the list of robust associations with an overall OR of 1.3 in the meta-analysis. A preliminary analysis by 23andMe using a recessive model for *PRKN* p.R275W, showed that in a heterozygous state OR is at 1.4, whereas in a homozygous alternative state the OR is 6. This variant is under heavy debate for its association with PD risk in a heterozygous state and is likely only disease causal if another damaging *PRKN* is on the other allele (Zhu et al. 2022).

Third, our analysis started with selection of variants based on array genotype data of 23andMe and therefore we missed variants that cannot be reliably imputed or are hard to genotype (such as *GBA1* p.L444P). This is a limitation that will be resolved with the availability of more sequencing data in the coming years.

Finally, the lack of diversity is a critical challenge in genetic research and limits our insights and understanding of the disease, for example current sample sizes in 23andMe for African and Asian ancestry is under 400 cases (Kim et al. 2022). However, initiatives such as the Global Parkinson’s Genetics Program (GP2) are making an active effort to increase the number of non-European underrepresented populations in genetic datasets (Global Parkinson’s Genetics Program 2021), so that in the near future, based on genetics, we can hopefully create a more representative picture of the disease.

In summary, we provide a robust list of 11 variants associated with PD and more reliable risk estimates. Additionally we provide a list of variants that were previously considered pathogenic and risk variants for PD which were not replicated in our analysis and should be treated with caution moving forward. Assessing rare variants is crucial to improve our understanding of disease development and heritability, but several complications arise when working with low frequency levels, such as lack of power. Larger data sets and more suitable tools are a critical requirement to further our understanding of rare variants, and we hope this work can be a useful tool for the wider PD community.

## Figures and Tables

**Table 1:** Cohort demographics

	23andMe	AMP-PD	UKB	Total
**Cases, n**	25,034	1,451	1,105 (+6,701 proxies)#	**27,590 (+6,701 proxies)**
Age, mean ± SD	72.3 (± 10.9)~	61.3 (±10.2)*	59.1 (± 7.1)^	
Male %	59.9	63.7	45.6	
**Controls**	3,065,473	2,556	38,051	**3,106,080**
Age, mean ± SD	50.1 (±17.5)	70.7 (±13.2)*	64.1 (±2.8)^	
Male %	43.5	49.8	48.6	
**Nominated variants**	692	301	644	
**Type of data**	Imputed genotype data	Whole genome sequencing	Whole exome sequencing	

UKB: UK Biobank, AMP-PD: Accelerating Medicines Partnership Parkinson’s disease

# proxies in UK Biobank data include: 6,033 parents and 668 siblings

~ Current age at time of analysis

* Age at analysis for AMP-PD

^ Age at recruitment for UK Biobank data

**Table 2:** List of 11 variants passing Bonferroni correction in meta-analysis.

rsID	Variant name	Ref	Alt	MAF 23andMe	MAF gnomAD	CLNSIG	Power OR 1.5	Power OR 3	Effect	SE	OR	L95% CI	U95% CI
rs80356771	GBA1 P.R502C	G	A	0.00019929	0	P	23%	96%	1.33	0.18	3.80	2.66	5.43
rs76763715	GBA1 P.N409S	T	C	0.00311143	0.0018	P	100%	100%	0.82	0.05	2.26	2.05	2.50
rs2230288	GBA1 P.E365K	C	T	0.01182293	0.0141	C/U/U	100%	100%	0.36	0.03	1.44	1.34	1.54
rs34637584	LRRK2 P.G2019S	G	A	0.00062618	3.00E-04	P/R	59%	100%	2.46	0.06	11.76	10.48	13.20
rs75548401	GBA1 P.T408M	G	A	0.00685015	0.0115	C/U/U	100%	100%	0.39	0.04	1.48	1.36	1.60
rs147138516	GBA1 P.D179H	C	G	0.00012406	1.00E-04	C/U/U	16%	82%	1.19	0.22	3.27	2.14	5.00
rs34995376	LRRK2 P.R1441H	G	A	3.47E-06	0	P	5%	100%	3.76	0.58	43.03	13.83	133.94
rs34424986	PRKN p.R275W	G	A	0.0024927	0.0033	P	99%	100%	0.26	0.06	1.30	1.15	1.47
rs111910483	LRRK2 p.L1795F	G	T	8.48E-05	NA	C/U/U	13%	100%	0.91	0.21	2.49	1.65	3.75
rs78973108	GBA1 P.R296Q	C	T	1.81E-05	1.00E-04	P	7%	*	1.31	0.29	3.71	2.12	6.49
rs758447515	GBA1 P.S146L	G	A	4.03E-07	NA	C/U/U	5%	*	7.39	1.70	1,624.73	58.44	45,172.33

Ref: reference allele, Alt: alternate allele, MAF: minor allele frequency, CLNSIG: clinical significance based on ClinVar, SE: standard error, L95% CI: Lower boundary 95% confidence interval, OR: odds ratio, U95% CI: upper boundary 95% confidence interval, P: pathogenic, P/R: pathogenic/risk factor, C/U/U: conflicting, uncertain, unknown.

* Minor allele frequencies too low for the tool to calculate statistical power at OR = 3 and alpha 0.05 in an additive model

# gnomAD frequencies based on NFE (non-finnish European)

**Variant name** indicates variants not previously associated with PD according to ClinVar

## Data Availability

Accelerating Medicines Partnership in Parkinson’s Disease (AMP PD data) and quality control notebooks are access-controlled [https://amp-pd.org/], and require individual sign-up to access the data. United Kingdom Biobank (UKBiobank) data are access-controlled and require an application for access [https://www.ukbiobank.ac.uk/]. Each contributing study abided by the ethics guidelines set out by their institutional review boards, and all participants gave informed consent for inclusion in both their initial cohorts and subsequent studies. All data produced in the present work are contained in the manuscript. All authors and the public can access the statistical programming code used in this project for the analyses and results generation. VP and CB take final responsibility for the decision to submit the paper for publication. Studies that are conducted on de-identified human genetics are waived ethical approval by the NIH Intramural IRB, as they are considered non-human subjects research.

## References

[1] Bandres-Ciga Sara, Diez-Fairen Monica, Kim Jonggeol Jeff, and Singleton Andrew B.. 2020. “Genetics of Parkinson’s Disease: An Introspection of Its Journey towards Precision Medicine.” Neurobiology of Disease 137 (April): 104782. 31991247 10.1016/j.nbd.2020.104782 PMC7064061

[2] Blauwendraat Cornelis, Kia Demis A., Pihlstrøm Lasse, Gan-Or Ziv, Lesage Suzanne, Gibbs J. Raphael, Ding Jinhui, 2018. “Insufficient Evidence for Pathogenicity of SNCA His50Gln (H50Q) in Parkinson’s Disease.” Neurobiology of Aging 64 (April): 159.e5–159.e8. 10.1016/j.neurobiolaging.2017.12.012 PMC5823280 29398121

[3] Blauwendraat Cornelis, Nalls Mike A., and Singleton Andrew B.. 2020. “The Genetic Architecture of Parkinson’s Disease.” Lancet Neurology 19 (2): 170–78. 31521533 10.1016/S1474-4422(19)30287-X PMC8972299

[4] Boyer David R., Li Binsen, Sun Chuanqi, Fan Weijia, Sawaya Michael R., Jiang Lin, and Eisenberg David S.. 2019. “Structures of Fibrils Formed by α-Synuclein Hereditary Disease Mutant H50Q Reveal New Polymorphs.” Nature Structural & Molecular Biology 26 (11): 1044–52. 10.1038/s41594-019-0322-y PMC6907165 31695184

[5] Brockmann Kathrin, Srulijes Karin, Pflederer Sylvia, Hauser Ann-Kathrin, Schulte Claudia, Maetzler Walter, Gasser Thomas, and Berg Daniela. 2015. “GBA-Associated Parkinson’s Disease: Reduced Survival and More Rapid Progression in a Prospective Longitudinal Study.” Movement Disorders: Official Journal of the Movement Disorder Society 30 (3): 407–11. 25448271 10.1002/mds.26071

[6] Bycroft Clare, Freeman Colin, Petkova Desislava, Band Gavin, Elliott Lloyd T., Sharp Kevin, Motyer Allan, 2018. “The UK Biobank Resource with Deep Phenotyping and Genomic Data.” Nature 562 (7726): 203–9. 30305743 10.1038/s41586-018-0579-z PMC6786975

[7] Chishti Muhammad A., Bohlega Saeed, Ahmed Maqbool, Loualich Arslan, Carroll Pamela, Sato Christine, St George-Hyslop Peter, Westaway David, and Rogaeva Ekaterina. 2006. “T313M PINK1 Mutation in an Extended Highly Consanguineous Saudi Family with Early-Onset Parkinson Disease.” Archives of Neurology 63 (10): 1483–85. 17030667 10.1001/archneur.63.10.1483

[8] Durand Eric Y., Do Chuong B., Mountain Joanna L., and Macpherson J. Michael. n.d. “Ancestry Composition: A Novel, Efficient Pipeline for Ancestry Deconvolution.” 10.1101/010512.

[9] Fuchs J., Nilsson C., Kachergus J., Munz M., Larsson E-M, Schüle B., Langston J. W., 2007. “Phenotypic Variation in a Large Swedish Pedigree due to SNCA Duplication and Triplication.” Neurology 68 (12): 916–22. 17251522 10.1212/01.wnl.0000254458.17630.c5

[10] Ghani Mahdi, Lang Anthony E., Zinman Lorne, Nacmias Benedetta, Sorbi Sandro, Bessi Valentina, Tedde Andrea, 2015. “Mutation Analysis of Patients with Neurodegenerative Disorders Using NeuroX Array.” Neurobiology of Aging 36 (1): 545.e9–14. 10.1016/j.neurobiolaging.2014.07.038 PMC4268030 25174650

[11] Global Parkinson’s Genetics Program. 2021. “GP2: The Global Parkinson’s Genetics Program.” Movement Disorders: Official Journal of the Movement Disorder Society 36 (4): 842–51. 33513272 10.1002/mds.28494 PMC9290711

[12] den Heijer Jonas M., Cullen Valerie C., Quadri Marialuisa, Schmitz Arnoud, Hilt Dana C., Lansbury Peter, Berendse Henk W., 2020. “A Large-Scale Full GBA1 Gene Screening in Parkinson’s Disease in the Netherlands.” Movement Disorders: Official Journal of the Movement Disorder Society 35 (9): 1667–74. 32618053 10.1002/mds.28112 PMC7540512

[13] Heilbron Karl, Jensen Melanie P., Bandres-Ciga Sara, Fontanillas Pierre, Blauwendraat Cornelis, Nalls Mike A., Singleton Andrew B., 2021. “Unhealthy Behaviours and Risk of Parkinson’s Disease: A Mendelian Randomisation Study.” Journal of Parkinson’s Disease 11 (4): 1981–93. 10.3233/JPD-202487 PMC8609708 34275906

[14] Henn Brenna M., Hon Lawrence, Macpherson J. Michael, Eriksson Nick, Saxonov Serge, Pe’er Itsik, and Mountain Joanna L.. 2012. “Cryptic Distant Relatives Are Common in Both Isolated and Cosmopolitan Genetic Samples.” PloS One 7 (4): e34267. 22509285 10.1371/journal.pone.0034267 PMC3317976

[15] Kalogeropulou Alexia F., Purlyte Elena, Tonelli Francesca, Lange Sven M., Wightman Melanie, Prescott Alan R., Padmanabhan Shalini, Sammler Esther, and Alessi Dario R.. 2022. “Impact of 100 *LRRK2* Variants Linked to Parkinson’s Disease on Kinase Activity and Microtubule Binding.” Biochemical Journal 479 (17): 1759–83. 35950872 10.1042/BCJ20220161 PMC9472821

[16] Khan Naheed L., Graham Elizabeth, Critchley Peter, Schrag Anette E., Wood Nicholas W., Lees Andrew J., Bhatia Kailash P., and Quinn Niall. 2003. “Parkin Disease: A Phenotypic Study of a Large Case Series.” Brain: A Journal of Neurology 126 (Pt 6): 1279–92. 12764051 10.1093/brain/awg142

[17] Kim Jonggeol Jeffrey, Vitale Dan, Otani Diego Véliz, Lian Michelle, Heilbron Karl, Iwaki Hirotaka, Lake Julie, 2022. “Multi-Ancestry Genome-Wide Meta-Analysis in Parkinson’s Disease.” medRxiv. 10.1101/2022.08.04.22278432.

[18] Lesage Suzanne, Lunati Ariane, Houot Marion, Romdhan Sawssan Ben, Clot Fabienne, Tesson Christelle, Mangone Graziella, 2020. “Characterization of Recessive Parkinson’s Disease in a Large Multicenter Study.” Annals of Neurology, May. 10.1002/ana.25787. PMC8944279 33045815

[19] Lubbe Steven J., Bustos Bernabe I., Hu Jing, Krainc Dimitri, Joseph Theresita, Hehir Jason, Tan Manuela, 2021. “Assessing the Relationship between Monoallelic PRKN Mutations and Parkinson’s Risk.” Human Molecular Genetics 30 (1): 78–86. 33448283 10.1093/hmg/ddaa273 PMC8033143

[20] Makarious Mary B., Lake Julie, Pitz Vanessa, Fu Allen Ye, Guidubaldi Joseph L., Solsberg Caroline Warly, Bandres-Ciga Sara, 2022. “Large-Scale Rare Variant Burden Testing in Parkinson’s Disease Identifies Novel Associations with Genes Involved in Neuro-Inflammation.” medRxiv. 10.1101/2022.11.08.22280168.

[21] Meeus Bram, Theuns Jessie, and Van Broeckhoven Christine. 2012. “The Genetics of Dementia with Lewy Bodies: What Are We Missing?” Archives of Neurology 69 (9): 1113–18. 22635379 10.1001/archneurol.2011.3678

[22] Moore Camille M., Jacobson Sean A., and Fingerlin Tasha E.. 2019. “Power and Sample Size Calculations for Genetic Association Studies in the Presence of Genetic Model Misspecification.” Human Heredity 84 (6): 256–71. 32721961 10.1159/000508558 PMC7666027

[23] Nalls Mike A., Blauwendraat Cornelis, Vallerga Costanza L., Heilbron Karl, Bandres-Ciga Sara, Chang Diana, Tan Manuela, 2019. “Identification of Novel Risk Loci, Causal Insights, and Heritable Risk for Parkinson’s Disease: A Meta-Analysis of Genome-Wide Association Studies.” Lancet Neurology 18 (12): 1091–1102. 31701892 10.1016/S1474-4422(19)30320-5 PMC8422160

[24] Nichols W. C., Elsaesser V. E., Pankratz N., Pauciulo M. W., Marek D. K., Halter C. A., Rudolph A., Shults C. W., Foroud T., and Parkinson Study Group-PROGENI Investigators. 2007. “LRRK2 Mutation Analysis in Parkinson Disease Families with Evidence of Linkage to PARK8.” Neurology 69 (18): 1737–44. 17804834 10.1212/01.wnl.0000278115.50741.4e

[25] Norton Edward C., Dowd Bryan E., and Maciejewski Matthew L.. 2018. “Odds Ratios-Current Best Practice and Use.” JAMA: The Journal of the American Medical Association 320 (1): 84–85. 29971384 10.1001/jama.2018.6971

[26] Paisán-Ruíz Coro, Jain Shushant, Evans E. Whitney, Gilks William P., Simón Javier, van der Brug Marcel, de Munain Adolfo López, 2004. “Cloning of the Gene Containing Mutations That Cause PARK8-Linked Parkinson’s Disease.” Neuron 44 (4): 595–600. 15541308 10.1016/j.neuron.2004.10.023

[27] Pawliczek Piotr, Patel Ronak Y., Ashmore Lillian R., Jackson Andrew R., Bizon Chris, Nelson Tristan, Powell Bradford, 2018. “ClinGen Allele Registry Links Information about Genetic Variants.” Human Mutation 39 (11): 1690–1701. 30311374 10.1002/humu.23637 PMC6519371

[28] Plaitakis Andreas, Latsoudis Helen, Kanavouras Konstantinos, Ritz Beate, Bronstein Jeff M., Skoula Irene, Mastorodemos Vasileios, 2010. “Gain-of-Function Variant in GLUD2 Glutamate Dehydrogenase Modifies Parkinson’s Disease Onset.” European Journal of Human Genetics: EJHG 18 (3): 336–41. 19826450 10.1038/ejhg.2009.179 PMC2987208

[29] Polymeropoulos M. H., Lavedan C., Leroy E., Ide S. E., Dehejia A., Dutra A., Pike B., 1997. “Mutation in the Alpha-Synuclein Gene Identified in Families with Parkinson’s Disease.” Science 276 (5321): 2045–47. 9197268 10.1126/science.276.5321.2045

[30] Proukakis Christos, Dudzik Christopher G., Brier Timothy, MacKay Donna S., Cooper J. Mark, Millhauser Glenn L., Houlden Henry, and Schapira Anthony H.. 2013. “A Novel α-Synuclein Missense Mutation in Parkinson Disease.” Neurology 80 (11): 1062–64. 23427326 10.1212/WNL.0b013e31828727ba PMC3653201

[31] Purcell Shaun, Neale Benjamin, Todd-Brown Kathe, Thomas Lori, Ferreira Manuel A. R., Bender David, Maller Julian, 2007. “PLINK: A Tool Set for Whole-Genome Association and Population-Based Linkage Analyses.” American Journal of Human Genetics 81 (3): 559–75. 17701901 10.1086/519795 PMC1950838

[32] Ray Soumya, Bender Samantha, Kang Stephanie, Lin Regina, Glicksman Marcie A., and Liu Min. 2014. “The Parkinson Disease-Linked LRRK2 Protein Mutation I2020T Stabilizes an Active State Conformation Leading to Increased Kinase Activity.” The Journal of Biological Chemistry 289 (19): 13042–53. 24695735 10.1074/jbc.M113.537811 PMC4036318

[33] Stoker Thomas B., Camacho Marta, Winder-Rhodes Sophie, Liu Ganqiang, Scherzer Clemens R., Foltynie Thomas, Evans Jonathan, Breen David P., Barker Roger A., and Williams-Gray Caroline H.. 2020. “Impact of Variants on Long-Term Clinical Progression and Mortality in Incident Parkinson’s Disease.” Journal of Neurology, Neurosurgery, and Psychiatry 91 (7): 695–702. 32303560 10.1136/jnnp-2020-322857 PMC7361014

[34] Szumilas Magdalena. 2010. “Explaining Odds Ratios.” Journal of the Canadian Academy of Child and Adolescent Psychiatry = Journal de l’Academie Canadienne de Psychiatrie de L’enfant et de L’adolescent 19 (3): 227–29. PMC2938757 20842279

[35] Wang Kai, Li Mingyao, and Hakonarson Hakon. 2010. “ANNOVAR: Functional Annotation of Genetic Variants from High-Throughput Sequencing Data.” Nucleic Acids Research 38 (16): e164. 20601685 10.1093/nar/gkq603 PMC2938201

[36] Wang Quanbao, Tian Qian, Song Xiaojie, Liu Yunyong, and Li Wei. 2016. “SNCA Gene Polymorphism May Contribute to an Increased Risk of Alzheimer’s Disease.” Journal of Clinical Laboratory Analysis 30 (6): 1092–99. 27184464 10.1002/jcla.21986 PMC6807080

[37] Willer Cristen J., Li Yun, and Abecasis Gonçalo R.. 2010. “METAL: Fast and Efficient Meta-Analysis of Genomewide Association Scans.” Bioinformatics 26 (17): 2190–91. 20616382 10.1093/bioinformatics/btq340 PMC2922887

[38] Zhan Xiaowei, Hu Youna, Li Bingshan, Abecasis Goncalo R., and Liu Dajiang J.. 2016. “RVTESTS: An Efficient and Comprehensive Tool for Rare Variant Association Analysis Using Sequence Data.” Bioinformatics 32 (9): 1423–26. 27153000 10.1093/bioinformatics/btw079 PMC4848408

[39] Zhu William, Huang Xiaoping, Yoon Esther, Bandres-Ciga Sara, Blauwendraat Cornelis, Billingsley Kimberly J., Cade Joshua H., 2022. “Heterozygous PRKN Mutations Are Common but Do Not Increase the Risk of Parkinson’s Disease.” Brain: A Journal of Neurology 145 (6): 2077–91. 35640906 10.1093/brain/awab456 PMC9423714

[40] Zimprich Alexander, Biskup Saskia, Leitner Petra, Lichtner Peter, Farrer Matthew, Lincoln Sarah, Kachergus Jennifer, 2004. “Mutations in LRRK2 Cause Autosomal-Dominant Parkinsonism with Pleomorphic Pathology.” Neuron 44 (4): 601–7. 15541309 10.1016/j.neuron.2004.11.005

